# Early postoperative physical activity and function: a descriptive case series study of 53 patients after lumbar spine surgery

**DOI:** 10.1186/s12891-020-03816-y

**Published:** 2020-11-27

**Authors:** Rogelio A. Coronado, Hiral Master, Daniel K. White, Jacquelyn S. Pennings, Mackenzie L. Bird, Clinton J. Devin, Maciej S. Buchowski, Shannon L. Mathis, Matthew J. McGirt, Joseph S. Cheng, Oran S. Aaronson, Stephen T. Wegener, Kristin R. Archer

**Affiliations:** 1 Department of Orthopaedic Surgery, Center for Musculoskeletal Research, Vanderbilt University Medical Center, 1215 21st Avenue South, Medical Center East – South Tower, Suite 4200, Nashville, TN 37232 USA; 2 Department of Physical Medicine and Rehabilitation, Osher Center for Integrative Medicine, Vanderbilt University Medical Center, Nashville, TN USA; 3 Department of Physical Therapy, University of Delaware, Newark, DE USA; 4 Steamboat Orthopaedic and Spine Institute, Steamboat Springs, CO USA; 5 Division of Gastroenterology, Hepatology, and Nutrition, Vanderbilt University Medical Center, Nashville, TN USA; 6 Department of Kinesiology, University of Alabama in Huntsville, Huntsville, AL USA; 7 Department of Neurological Surgery, Carolina Neurosurgery and Spine Associates, Charlotte, NC USA; 8 Department of Neurological Surgery, University of Cincinnati College of Medicine, Cincinnati, OH USA; 9 Howell Allen Clinic, Saint Thomas Medical Partners, Nashville, TN USA; 10 Department of Physical Medicine and Rehabilitation, Johns Hopkins University, Baltimore, MD USA

**Keywords:** Walking, Physical activity, Postoperative period, Spinal stenosis, Spinal fusion

## Abstract

**Background:**

The purpose of this prospective case series study was to compare changes in early postoperative physical activity and physical function between 6 weeks and 3 and 6 months after lumbar spine surgery.

**Methods:**

Fifty-three patients (mean [95% confidence interval; CI] age = 59.2 [56.2, 62.3] years, 64% female) who underwent spine surgery for a degenerative lumbar condition were assessed at 6 weeks and 3- and 6-months after surgery. The outcomes were objectively-measured physical activity (accelerometry) and patient-reported and objective physical function. Physical activity was assessed using mean steps/day and time spent in moderate to vigorous physical activity (MVPA) over a week. Physical function measures included Oswestry Disability Index (ODI), 12-item Short Form Health Survey (SF-12), Timed Up and Go (TUG), and 10-Meter Walk (10 MW). We compared changes over time in physical activity and function using generalized estimating equations with robust estimator and first-order autoregressive covariance structure. Proportion of patients who engaged in meaningful physical activity (e.g., walked at least 4400 and 6000 steps/day or engaged in at least 150 min/week in MVPA) and achieved clinically meaningful changes in physical function were compared at 3 and 6 months.

**Results:**

After surgery, 72% of patients initiated physical therapy (mean [95%CI] sessions =8.5 [6.6, 10.4]) between 6 weeks and 3 months. Compared to 6 weeks post-surgery, no change in steps/day or time in MVPA/week was observed at 3 or 6 months. From 21 to 23% and 9 to 11% of participants walked at least 4400 and 6000 steps/day at 3 and 6 months, respectively, while none of the participants spent at least 150 min/week in MVPA at these same time points. Significant improvements were observed on ODI, SF-12, TUG and 10 MW (*p* <  0.05), with over 43 to 68% and 62 to 87% achieving clinically meaningful improvements on these measures at 3 and 6 months, respectively.

**Conclusion:**

Limited improvement was observed in objectively-measured physical activity from 6 weeks to 6 months after spine surgery, despite moderate to large function gains. Early postoperative physical therapy interventions targeting physical activity may be needed.

## Background

Over the last two decades there has been a considerable increase in the number of lumbar spine surgeries for degenerative conditions such as spinal stenosis [1–4]. One of the major contributors to this increase is the growing population of older adults [5]. From 2000 to 2010, older adults were the fastest growing population in the United States [6]. Over a similar 10-year time span, lumbar spine fusion rates increased 137% with aggregate annual hospital costs increasing nearly 8-fold to $34 billion [7, 8]. Despite the increased prevalence and surgical resources directed towards degenerative lumbar conditions, studies have reported that up to 40% of individuals report chronic pain or disability [9–12].

Physical function is a common outcome after spine surgery and can be assessed using patient-reported questionnaires and performance-based tests [13]. In healthy older adults, age-related declines in physical function are well documented; hence recovery of function is important in these individuals after spine surgery [14, 15]. The results of physical function measures are used to infer the ability of a patient to engage in physical activity in daily life. However, these measures may not reflect levels of physical activity such as amount or intensity of walking. This is important as a common assumption is that recovery of function co-occurs with greater engagement in health-promoting behaviors like walking.

There is a need to directly examine physical activity after lumbar spine surgery [16], especially since physical activity is a significant risk factor of general and pain-related disability [17–20]. Smuck et al. [21] compared preoperative and 6-month postoperative objective physical activity and function after spine surgery and found no significant changes in physical activity despite improvements in physical function. Schulte et al. [22] reported improvement in objectively-measured physical activity from preoperative to 3 and 12 months after decompression surgery for spinal stenosis, however the magnitude of physical activity change was less pronounced than patient-reported physical function. Mancuso and colleagues [23] reported on persistent patient-reported physical activity deficits, with their data demonstrating that only 26% of patients met recommended physical activity levels (e.g., ≥ 150 min/week of moderate activity) at 24 months after spine surgery. Gilmore et al. [24] found that patients who spent more time walking, as measured through accelerometry, during the first postoperative week after spine surgery have substantial improvement on disability at 6 months. While prior work suggests that spine surgery results in minimal physical activity benefit, little is known about changes in physical activity that may occur over the early postoperative period.

Postoperative physical therapy (PT) is often prescribed to patients within the first 3 months of spine surgery [25]. The main goals of postoperative PT are to help patients manage postoperative pain, increase muscle strength, and improve physical function. Overall, limited and low quality evidence supports the effectiveness of PT after spine surgery [26–29]. However, the early recovery period may be the optimal time-frame to promote physical activity, alongside physical function. A lack of improvement in physical activity, despite gains in physical function, may be an important finding to inform early postoperative PT. Additionally, there are few studies that have examined how patient demographic or clinical characteristics influence objectively-measured physical activity after spine surgery [22, 30]. Preliminary evidence has shown factors such as age, body mass index (BMI), and surgery type (i.e., fusion) may influence physical activity in a spine surgery population [23, 30]. Knowledge of potential moderating factors can inform targeted rehabilitation strategies to improve physical activity after spine surgery.

The primary purpose of this prospective case series study was to compare changes in early postoperative physical activity and physical function between 6 weeks and 3 and 6 months after spine surgery in patients with lumbar degenerative conditions. We also aimed to explore changes in early postoperative physical activity by important patient and clinical characteristics of age, sex, BMI, previous spine surgery, fusion status, and number of PT visits. We build on existing physical activity evidence that has explored similar factors in the immediate postoperative period (e.g., 1 week after surgery) and from preoperative to 12 months. These factors are also important covariates in spine surgery predictive models for patient-reported ouctomes [31]. No study has explored whether PT participation moderates physical activity after spine surgery.

## Methods

### Study design

This was a prospective case series study of patients undergoing surgery for a lumbar degenerative condition at a single academic medical center. Postoperative assessments were conducted at 6 weeks, 3 and 6 months after lumbar spine surgery. The Institutional Review Board at Vanderbilt University Medical Center provided ethical approval for this study.

### Participants

We enrolled patients undergoing surgery for a lumbar degenerative condition. Patients were considered eligible if they met the following criteria: 21 years of age or older; English-speaking; reported the presence of back and/or lower extremity pain greater than 6 months; diagnosed by a surgeon with a degenerative condition (i.e., spinal stenosis, spondylosis with or without myelopathy, degenerative spondylolisthesis); and treated surgically with a laminectomy with or without fusion.

Patients were excluded based on the following: spinal deformity as the primary indication for surgery; surgery for pseudarthrosis, trauma, infection, or tumor; microsurgical techniques or radicular symptoms caused by a prolapsed or sequestered disc; medical history of neurological disorder or disease resulting in moderate to severe movement dysfunction; medical history of schizophrenia or other psychotic disorder; surgery under workman’s compensation claim; and unable to return to clinic for follow-up visits with the surgeon. Patients undergoing revision surgery were excluded.

### Procedures

Eligible patients were approached at a standard preoperative clinic visit for screening and enrollment. After providing written informed consent, patients completed a preoperative demographic questionnaire for age, sex, height, weight, race, education level, marital status, smoking status, comorbidities, and history of previous spine surgery. Clinical characteristics such as fusion surgery (fusion, no fusion) and the number of PT visits from 6 weeks to 3 months were based on patient report through interviews and a web-based survey. At 6 weeks and 3 and 6 months after surgery, patients wore an accelerometer on the right hip for seven consecutive days during waking hours. Patients also completed patient-reported and objective physical function tests at in-person clinic visits.

### Tests and measures

#### Physical activity

Physical activity was assessed using a commercially available triaxial accelerometer (ActiGraph GT3X+, ActiGraph LLC, Pensacola, FL) [32], which assesses acceleration in a vertical, horizontal and perpendicular axis. Accelerometers are reliable and valid objective measures for quantifying physical activity in terms of steps/day and intensity in free-living adults [33–35]. We collected three-dimensional acceleration over 60-s epochs. Each patient was fitted with an accelerometer around the waist and instructed on how to attach the monitor at home. Patients were instructed to wear the accelerometer each morning after waking from sleep and remove it at bedtime for up to 7 consecutive days. Patients could remove the accelerometer for bathing, sleeping at night, or swimming. We modified a standardized method from the National Cancer Institute for determining valid wear time [36, 37]. Non-wear time was defined as activity counts less than 100 for 180 consecutive minutes [36, 38]. Accelerometer data with at least 10 h of wear time within a 24-h period was considered a valid day of monitoring [36, 37]. We limited our sample to participants who had a minimum of 4 days of valid wear time data as this is the minimum number of days needed for a reliable estimate of physical activity [39, 40]. Physical activity was measured in units of total volume of physical activity (mean steps/day) and intensity (mean time spent in moderate-to-vigorous physical activity [MVPA] over a day). National Cancer Institute intensity thresholds were applied to classify accelerometer counts into MVPA (> 2019 counts) on a minute-by-minute basis. We summed total daily time in minutes in MVPA. Walking at least 4400 steps/day and 6000 steps/day are associated with lower risk of mortality in older women [41] and developing functional limitation in adults with knee osteoarthritis [42], respectively. National Physical Activity guidelines for Americans recommend at least 150 min/week in MVPA to achieve health benefits [43]. Therefore, we used these thresholds for both steps/day and MVPA to investigate whether participants engaged in meaningful physical activity following spine surgery.

#### Physical function

##### Oswestry disability index

The 10-item Oswestry Disability Index (ODI) was used to assess condition-specific disability [44]. The ODI has good test-retest reliability, validity and internal consistency in chronic back pain and spine surgery populations [44, 45]. The minimal clinically important difference (MCID) for the ODI is a 30% reduction [46].

##### 12-item short form health survey

The physical component scale of 12-item Short Form Health Survey (SF-12) was used to assess general physical health. The physical component scale assesses four domains of physical functioning, role-physical, bodily pain, and general health. The physical component scale of the SF-12 has demonstrated good responsiveness, test–retest reliability, internal consistency, and validity in healthy and patient populations [47, 48]. The MCID for SF-12 is 3.3 points [49].

##### Timed up and go

The Timed Up and Go (TUG) was used to assess functional mobility. Patients were asked to stand from a standard armchair with a seat height of approximately 48 cm and arm height of approximately 65 cm. Patients walked 3 m, turned around, and walked back and sat down. The time in seconds to complete the TUG was recorded. The TUG has excellent test-retest reliability [50] and is moderate-to-strongly correlated with other functional mobility measures [51]. The MCID for the TUG is 1.3 s [52, 53].

##### 10-meter walk

The 10-Meter Walk (10 MW) was used to assess comfortable walking speed. The 10 MW was set up in hallway that allowed a 5-m warm-up distance, a 10-m test distance and then another 5 m beyond the 10 m for patients to continue walking. Patients were instructed to walk the 10 m at a comfortable pace. Two trials were conducted with a brief rest as needed between trials. An average time in seconds was computed and converted to walking speed (meters per second [m/s]). The 10 MW has excellent reliability [54, 55] and is correlated with measures of function and mortality in older adults [56, 57]. The MCID for the 10 MW is 0.08 m/s [58].

### Data analysis

Descriptive statistics were generated for demographic and clinical characteristics. To compare change in physical activity and function at 6 weeks, 3 months, and 6 months, we conducted separate generalized estimating equations (GEE) with robust estimator and first-order autoregressive covariance structure. GEE was performed for each outcome with time as a repeated measure. Pairwise comparisons with Bonferroni correction were examined between each time point. Multiple imputation using five imputed datasets and predictive mean matching was used to handle missing data [59]. Estimated marginal means, 95% confidence interval (CI), and *p* values were obtained from pooled effect estimates. We explored steps/day by our stratified variables of age (< 60 years, ≥ 60 years), sex, BMI (in kilogram/meter^2^ [kg/m^2^] < 24.9, 25.0–29.9, ≥ 30.0), previous spine surgery, fusion status, and PT visits and present estimated marginal means, 95% CIs, and *p*-values from separate GEE models. We also compared the proportion of adults who walked at least 4400 or 6000 steps/day, engaged in at least 150 min/week in MVPA, and achieved MCID for physical function measures at 3 and 6 months after spine surgery. Statistical significance was set at the 0.05 level. IBM SPSS Statistics for Windows, Version 27.0 (Armonk, NY: IBM Corp.) was used for all analyses.

## Results

### Participants

Data from 53 participants (mean [95% CI] age = 63.1 [59.7, 66.4] years; body mass index = 31.2 [28.4, 33.9] kg/m^2^ and 34 (64%) females) were examined (Table 1). After surgery, 72% of the participants initiated PT with a mean [95% CI] of 8.5 [6.6, 10.4] visits from 6 weeks to 3 months following spine surgery. Eighteen (34%) participants had previous spine surgery and 36 (68%) participants underwent fusion surgery.

**Table 1:** Demographic and clinical characteristics of enrolled participants (*N* = 53)

Characteristic	Mean (95% CI) or n (%)
Age, in years	63.1 (59.7, 66.4)
Female	34 (64%)
White	43 (81%)
Private Insurance	31 (58%)
> High school education	39 (74%)
Married	38 (72%)
Current Smoker	7 (13%)
At least 1 Comorbidity	50 (94%)
Body mass index, kg/m^2^	31.2 (28.4, 33.9)
Prior spine surgery	18 (34%)
Fusion performed	36 (68%)
Number of physical therapy visits	8.5 (6.6, 10.4)

### Physical activity

Participants wore the accelerometer for a mean [95% CI] of 15.2 [14.6, 15.7] hours/day at 6 weeks, 15.0 [14.5, 15.6] hours/day at 3 months, and 15.2 [14.6, 15.7] hours/day at 6 months (Table 2). At these same time points, participants achieved a mean [95% CI] of 3310 [2776, 3844] steps/day, 3705 [3292, 4119] steps/day, and 3781 [3289, 4273] steps/day, respectively. No significant change over time was observed (*p* = 0.07). Participants spent 0 min/week in MVPA at 6 weeks and 3 and 6 months after surgery. Twenty-three percent and 21% percent of participants walked at least 4400 steps/day and 9 and 11% of participants walked at least 6000 steps/day at 3 and 6 months after spine surgery, respectively (Table 3). No participants were able to achieve the recommended time of 150 min/week in MVPA at 3 and 6 months after spine surgery.

**Table 2:** Change in early postoperative physical activity and function from 6 weeks to 3 and 6 months after lumbar spine surgery

Outcome Variable	6 weeks Mean (95% CI)	3 months Mean (95% CI)	6 months Mean (95% CI)	***p***-value*
***Physical activity***				
Wear time (hours/day)	15.2 (14.6; 15.7)	15.0 (14.5; 15.6)	15.2 (14.6; 15.7)	0.81
Steps/day	3310 (2776; 3844)	3705 (3292; 4119)	3781 (3289; 4273)	0.07
Time in MVPA (minutes/week)	0	0	0	–
***Physical function***				
ODI	34.7 (30.0; 39.4)	26.1 (21.3; 30.9)	21.6 (16.7; 26.4)	< 0.05
SF-12 Physical Health	30.5 (27.6; 33.4)	38.0 (35.0; 40.9)	42.4 (39.4; 45.4)	< 0.05
TUG (s)	11.2 (10.0; 12.4)	10.4 (9.6; 11.3)	9.8 (9.0; 10.7)	< 0.05
10 MW (m/s)	1.04 (0.96; 1.12)	1.12 (1.05; 1.19)	1.19 (1.10; 1.27)	< 0.05

*p-value obtained from pooled generalized estimating equations and indicates a statistical difference between at least two of the time points

Abbreviations: *10 MW* 10-Meter Walk; *m/s* Meters per second; *MVPA* Moderate to vigorous physical activity; *ODI* Oswestry Disability Index; *s* Seconds; *SF-12* 12-Item Short-Form Health Survey; *TUG* Timed Up and Go

**Table 3:** Number and percentage of participants achieving recommendations for physical activity and meaningful change in physical function at 3 and 6 months after spine surgery

Outcome Variable	n (%) at 3 months	n (%) at 6 months
***Physical Activity***		
Achieving > 4400 steps/day	12 (23%)	11 (21%)
Achieving > 6000 steps/day	5 (9%)	6 (11%)
Achieving > 150 min/week in MVPA	0	0
***Physical Function***		
Change in ODI > 30%	23 (43%)	33 (62%)
Change in SF-12 Physical Health > 3.3	36 (68%)	46 (87%)
Change in TUG > 1.3 s	34 (64%)	33 (62%)
Change in 10 MW > 0.08 m/s	24 (45%)	34 (64%)

Abbreviations: *10 MW* 10-Meter Walk; *m/s* Meters per second; *MVPA* Moderate to vigorous physical activity; *ODI* Oswestry Disability Index; *s* Seconds; *SF-12* 12-Item Short-Form Health Survey; *TUG* Timed Up and Go

### Patient-reported physical function

**Fig. 1:**
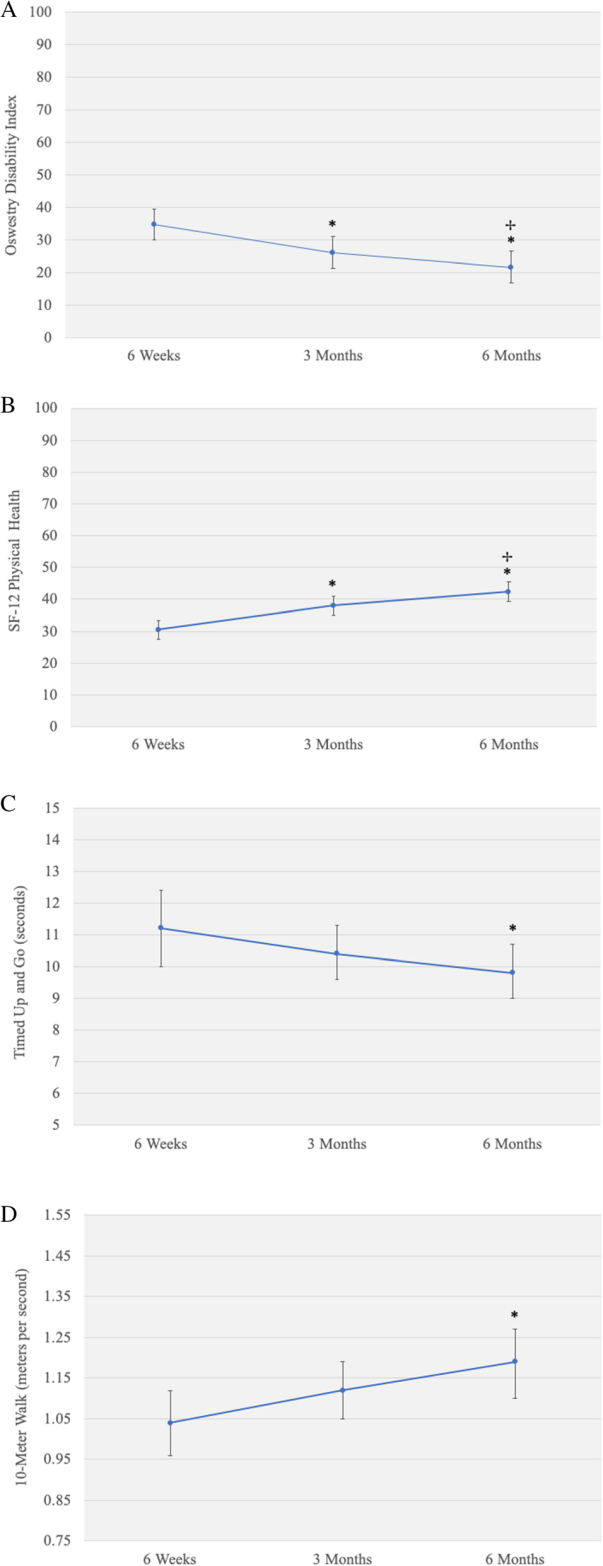
Change in early postoperative patient-reported outcomes (**a** and **b**) and objective physical function (**c** and **d**). Error bars are 95% confidence intervals. * indicates significant difference from 6 weeks. ^✢^ indicates significant difference from 3 months

ODI scores were 34.7 [30.0, 39.4] at 6 weeks, 26.1 [21.3, 30.9] at 3 months, and 21.6 [16.7, 26.4] at 6 months (Table 2). At these same time points, SF-12 physical health scores were 30.5 [27.6, 33.4], 38.0 [35.0, 40.9], and 42.4 [39.4, 45.4]. There were significant improvements over time for ODI and SF-12 physical health (*p* <  0.05) (Fig. 1a and b). Compared to 6 weeks after surgery, significant reductions in ODI were observed at 3 (mean change [95% CI] = − 8.6 [− 11.0, − 6.2]) and 6 months after surgery (mean change [95% CI] = − 13.1 [− 16.3, − 9.9]). Compared to 6 weeks after surgery, significant increases in SF-12 physical health were observed at 3 (mean change [95% CI] = 7.5 [5.4, 9.5]) and 6 months after surgery (mean change [95% CI] = 11.9 [9.3, 14.4]). Forty-three percent and 62% of the sample achieved MCID change on the ODI at 3 and 6 months after spine surgery, respectively, while 68 and 87% of the sample achieved MCID change on the SF-12 at same time points (Table 3).

### Objective physical function

Time needed to complete the TUG test was 11.2 [10.0, 12.4] seconds at 6 weeks, 10.4 [9.6, 11.3] seconds at 3 months, and 9.8 [9.0, 10.7] at 6 months (Table 2). At these same time points, walking speed on 10 MW test was 1.04 [0.96, 1.12] m/s, 1.12 [1.05, 1.19] m/s, and 1.19 [1.10, 1.27] m/s. There was a significant improvement over time for the TUG and 10 MW (*p* <  0.05) (Fig. 1c and d). Compared to 6 weeks after surgery, there was a significant decrease at 6 months in the time needed to complete the TUG (mean change [95% CI] = − 1.3 [− 2.3, − 0.3]) and in increase at 6 months for 10 MW speed (mean change [95% CI] = 0.15 [0.05, 0.25]). Sixty-four percent and 62% of the sample achieved MCID change on the TUG at 3 and 6 months after spine surgery, respectively, while 45 and 64% of the sample achieved MCID change on the 10 MW at the same timepoints (Table 3).

### Exploration of physical activity stratified by demographic and clinical characteristics

Similar steps/day values over time were observed based on age, sex, fusion status, and PT visits (Table 4). There was a difference noted in steps/day over time based on BMI (*p* <  0.05). The low BMI group (< 24.9 kg/m^2^) had higher steps/day than the other two BMI groups at 6 weeks and 6 months but not at 3 months after surgery. There was also a difference in steps/day over time based on previous spine surgery (*p* <  0.05). Patients without a previous spine surgery had higher steps/day at 6 months compared to 6 weeks, whereas no difference in steps/day over time was noted in the previous spine surgery group.

**Table 4:** Description of steps/day by demographic and clinical characteristics

		6 weeks	3 months	6 months	***p***-value*
Characteristic	n	Mean (95% CI)	Mean (95% CI)	Mean (95% CI)	
***Age***					0.81
< 60 years	25	3444 (2767; 4121)	3732 (3225; 4239)	3766 (3086; 4444)	
≥ 60 years	28	3191 (2383; 3998)	3681 (3044; 4319)	3794 (3088; 4500)	
***Sex***					0.35
Male	19	3986 (3105; 4867)	4170 (3391; 4948)	4050 (3233; 4867)	
Female	34	2933 (2296; 3569)	3446 (2994; 3897)	3630 (3020; 4241)	
***BMI***					< 0.05
< 24.9 kg/m^2^	10	5470 (4215; 6724)	4652 (3668; 5636)	5472 (4463; 6480)	
25–29.9 kg/m^2^	16	3220 (2405; 3935)	3726 (3030; 4421)	3357 (2554; 4260)	
≥ 30 kg/m^2^	27	2564 (1950; 3178)	3343 (2805; 3881)	3405 (2839; 3972)	
***Previous spine surgery***					< 0.05
No	35	3374 (2651; 4097)	3801 (3327; 4275)	4178 (3579; 4777)	
Yes	18	3186 (2486; 3887)	3519 (2733; 4306)	3008 (2267; 3750)	
***Surgery type***					0.38
No fusion	17	3667 (2695; 4640)	3899 (3173; 4625)	3716 (3089; 4343)	
Fusion	36	3141 (2511; 3772)	3614 (3114; 4114)	3811 (3150; 4472)	
***PT visits***					0.20
> 10 visits	21	3724 (2809; 4639)	3893 (3250; 4536)	3701 (2993; 4410)	
< 10 visits	32	3039 (2385; 3693)	3583 (3044; 4121)	3833 (3158; 4508)	

*p-value obtained from pooled generalized estimating equations and indicates a statistical difference in physical activity based on characteristic

Abbreviations: *BMI* Body mass index; *CI* Confidence interval; *PT* Physical therapy

## Discussion

The primary purpose of this study was to compare changes in early postoperative physical activity and physical function between 6 weeks and 3 and 6 months after spine surgery in patients with lumbar degenerative conditions. The findings of this study demonstrated that patients engage in very low levels of physical activity over the early postoperative period, both in terms of steps/day and minutes/week in MVPA after lumbar spine surgery. Overall, we did not observe a change over time in physical activity from 6 weeks to 3 and 6 months after lumbar spine surgery, which was in contrast to large significant improvements in physical function over the same time period. This observation was also supported by a smaller proportion of patients achieving meaningful thresholds for physical activity compared to physical function. We found preliminary evidence that BMI and previous spine surgery may be important factors that may influence postoperative physical activity patterns.

There are a few studies describing physical activity after lumbar spine surgery [21–23, 60, 61]. Rolving et al. [61] examined patient-reported physical activity using the Physical Activity Scale in patients 1 year after lumbar spine surgery and found patients reported a moderate level of physical activity based on self-report of activity time. The difference in mode of measuring physical activity (e.g., objectively measured vs. patient-reported) makes direct comparisons difficult. For example, a previous meta-analysis has shown overestimation of physical activity is a potential source of bias with patient-reported physical activity measures [62]. Similarly, a study by Mancuso et al. [23] found 26% of patients meet the recommended time for MVPA at 2 years, which was higher compared to this study, where no participants met the recommended time at 6 months. Compared to the current study, Mancuso et al. [23] used a patient-reported physical activity measure as opposed to an objective measurement and the time of follow-up following spine surgery was longer (2 years vs. 6 months following spine surgery).

Schulte et al. [22] examined physical activity, measured by an ankle-worn activity monitor, preoperatively and at 3 and 12 months after surgery. At the 3-month time point, Schulte et al. [22] reported patients achieved an average of 4145 gait cycles per day, which is approximately a two-fold higher physical activity level compared to the current study (i.e., 1 gait cycle = 2 steps). A study conducted in Japan by Inoue et al. [60] found that the activity counts increased at 3-, 6- and 12-months following spine surgery compared to the preoperative time point. One potential explanation for differences in objectively-measured physical activity levels may relate to the geographic region (i.e., culture) and sample characteristics. The patients in the current study were younger and had a slightly higher and more variable BMI than the sample by Schulte et al. [22], and had higher BMI than the sample by Inoue et al. [60]. The variability in BMI could be an influential factor in why we observed low values of physical activity. Additionally, the current sample included a greater proportion of patients undergoing fusion surgery and with a history of previous spine surgery. Another reason for physical activity differences across studies could be due to the monitoring devices and location of placement. Schulte et al. [22] used a StepWatch monitor worn on the ankle, Inoue et al. [60] used an Actigraph monitor worn on the wrist, and the current study used an Actigraph monitor worn around the hip. Previous studies in older adults have shown that wrist [63] and ankle [64] worn monitors may count more steps compared to hip worn monitors. Therefore, caution should be taken when comparing activity levels between studies that use different monitor placement.

The current study found no change in physical activity during the early postoperative period (6 weeks) to 3 and 6 months after surgery. These findings are consistent with prospective physical activity data reported in other studies of lumbar spine surgery [21, 22] and lower extremity arthroplasty [65, 66]. Schulte et al. [22] and Smuck et al. [21] observed limited change in postoperative physical activity from 3 to 12 months after lumbar spine surgery and from preoperative to 6 months after surgery, respectively. Similarly, in patients after total hip or knee arthroplasty, Harding et al. [66] found no change in physical activity from preoperative to 6 months and de Groot et al. [65] found no change from 3 to 6 months after surgery. In contrast to physical activity, physical function demonstrated medium to large changes after lumbar spine surgery. Changes in patient-reported physical function after lumbar spine surgery are well documented [67]. However, to date, few studies have documented changes in objectively-measured physical function [21, 68, 69]. Our results coincide with findings from Smuck et al. [21] showing that improvements in objectively-measured physical function do not translate to change in real-world physical activity.

In our exploratory analysis, we found no difference in physical activity over time based on PT participation. Coupled with the finding that most patients in our cohort attended PT, these results suggest standard PT is limited in altering physical activity following orthopaedic surgery. While postoperative education on physical activity is considered an important component of early postoperative care [29], several surveys have reported variable patterns on the type or extent of education or instruction on physical activity or exercise [25, 70, 71]. Structured behavioral strategies which target physical activity may need to be integrated within PT or provided as an adjunct strategy. For example, community-based interventions such as in-person counseling or group sessions have been shown to be effective in promoting physical activity, especially in older adults [72–74]. Future research should aim to establish feasible intervention strategies to improve physical activity after spine surgery.

In addition to PT participation, we explored whether other characteristics influenced postoperative physical activity. In the current study, BMI and previous spine surgery were observed to be potential moderators of physical activity change. Schulte et al. [22] assessed whether physical activity after surgery was affected by similar characteristics such as age, sex, BMI, previous spine surgery, and surgery type. In contrast to our findings, Schulte et al. [22] did not find an association between any of these characteristics and physical activity. Mancuso et al. [23] reported that higher BMI was one characteristic that was associated with not meeting recommended guidelines for physical activity 2 years after spine surgery. Similar to the current study, Mancuso et al. [23] did not find an association with fusion status. However, they did note associations with clinical variables not examined in the current study including number of surgical levels and having a degenerative disease. Gilmore et al. [30] found step counts within the first week after spine surgery differed based on factors such as age and surgery type. For example, patients who were older and underwent fusion surgery had less step counts during the initial postoperative week. There is a clear need for additional examination of potential factors that can assist in identifying postoperative spine patients at-risk for low physical activity during the recovery period.

The study has a few limitations to note. This was a small case series study intended to generate preliminary descriptive data on the levels of physical activity compared to physical function immediately after spine surgery. Due to the small sample, we are limited in our analysis strategy, especially in performing multivariable analyses to account for additional covariates that may influence physical activity. Larger studies would be needed to validate our observations. We did not control for varying types of postoperative advice, education, or physical therapy patients may have received. Most of our enrolled patients received physical therapy, however, we do not have data on the specific types of therapeutic activities.

## Conclusions

These results demonstrate low levels of physical activity over the early postoperative period in patients after lumbar spine surgery for degenerative conditions. Little to no improvement was observed in objectively measured physical activity from 6 weeks to 6 months after spine surgery, despite moderate to large physical function gains.

## Data Availability

The datasets used and/or analyzed during the current study are available by reasonable request and at the discretion of the corresponding author.

## Abbreviations

10 MW: 10-Meter Walk
ANOVA: Analysis of variance
BMI: Body mass index
m/s: Meters per second
MCID: Minimal clinically important difference
MVPA: Moderate to vigorous physical activity
ODI: Oswestry Disability Index
PT: Physical therapy
s: Seconds
SF-12: 12-Item Short-Form Health Survey
TUG: Timed Up and Go
